# Pericardial agenesis

**DOI:** 10.1007/s10554-025-03488-6

**Published:** 2025-08-07

**Authors:** Aaroh K. Patel, Leila Rezai Gharai

**Affiliations:** 1 Virginia Commonwealth University School of Medicine (A.K.P), 1201 E Marshall St #4-100, Richmond, VA 23298 USA; 2 Department of Radiology (L.R.G), Virginia Commonwealth University Health System, 1200 E Marshall St, Richmond, VA 23298 USA

**Keywords:** Pericardial agenesis, Magnetic resonance imaging, Levoposition

## Abstract

We report a case of complete pericardial agenesis (PA). PA is a rare congenital anomaly characterized by complete or partial defects in the pericardium [1]. Cardiac magnetic resonance imaging (MRI) is key in diagnosing this condition.

## Main body

**Fig. 1:**
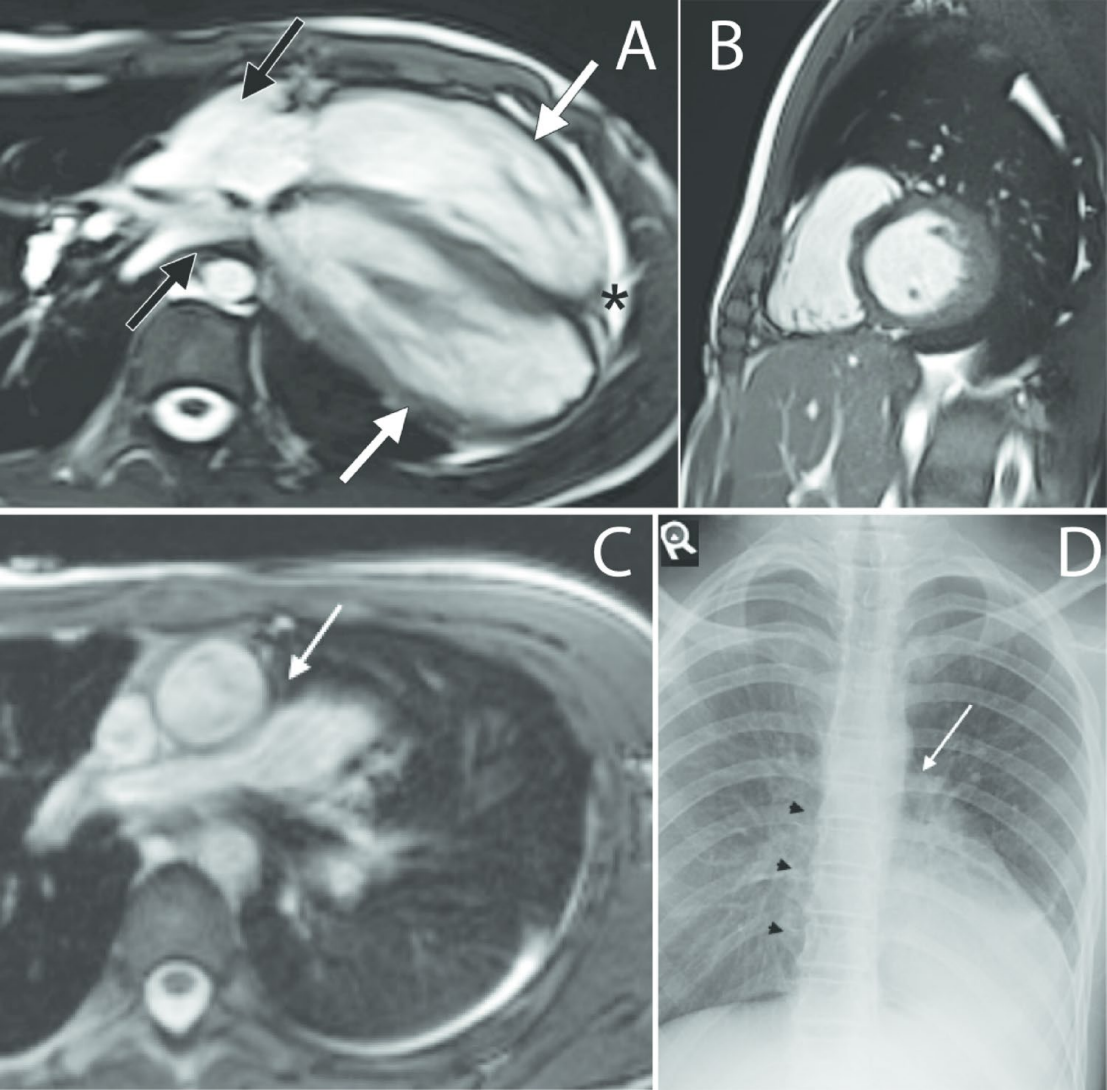
(**A**) Cardiac MRI SSFP cine image in four-chamber view shows elongated appearance of both atria (black arrows) and relatively dilated ventricles (white arrows) giving tear drop morphology. The pericardium is not seen in the fat surrounding the heart (*). (**B**) Cardiac MRI Steady State Free Precession (SSFP) cine image in short-axis view shows a portion of the left lung (black arrow) insinuate below the inferior wall of the left ventricle (white arrow) during the cardiac cycle. (**C**) Cardiac MRI SSFP cine image in axial view showing interposition of lung tissue between the ascending aorta and main pulmonary artery (white arrow). (**D**) The chest X-ray demonstrates straightening and elongation of the left cardiac border, loss of right cardiac border (black arrow heads), and interposition of pulmonary tissue between the aortic knob and main pulmonary artery (white arrow)

A 13-year-old male was admitted for a non-cardiac medical condition. During evaluation, echocardiography revealed cardiac levoposition. Cardiac MRI was requested to assess morphology and function. Imaging depicted leftward cardiac displacement with atrial elongation and widening of the ventricles (Fig. 1A). Lung tissue was interposed between the left ventricle (LV) and diaphragm (Fig. 1B). The pericardium was not visualized. Absence of past-surgical history and imaging ruled out differentials of post-surgical shift and mediastinal mass effect. A diagnosis of complete PA was established. No intervention was initiated as the pericardial anomaly was deemed noncontributory to the patient’s presentation.

On MRI, the pericardium is best visualized with cine-steady-state free-procession or axial fast-spin-echo sequences with 4–8-millimeter slices. The pericardium is best seen along the right ventricular wall but poorly visualized along the LV lateral wall [1]. As non-visualization along the LV is not specific, diagnosis requires ancillary imaging findings.

PA is typically asymptomatic but can be associated with congenital anomalies [2]. Management of PA is controversial given its rarity. Most asymptomatic patients are managed conservatively; however, surgical intervention may be pursued in symptomatic patients or defects complicated by cardiac chamber herniation or vascular narrowing, particularly of the coronary arteries and pulmonary veins [3].

## Data Availability

No datasets were generated or analysed during the current study.
